# Analysis of Cerebrospinal Fluid Routine Biochemical Level, Pathogenic Bacteria Distribution, and Risk Factors in Patients with Secondary Intracranial Infection after Brain Tumor Surgery

**DOI:** 10.1155/2022/7716205

**Published:** 2022-09-16

**Authors:** Yang Zhang, Ying Zhou, Min Hou, Sunfu Zhang

**Affiliations:** ^1^Department of Neurosurgery, The Third People's Hospital of Chengdu, Chengdu, Sichuan 610031, China; ^2^Department of Cosmetic Minimally Invasive, Sichuan Huamei Zixin Medical Cosmetology Hospital, Chengdu, Sichuan 610000, China

## Abstract

**Purpose:**

Analysis of routine biochemical levels of cerebrospinal fluid (CSF), distribution of pathogenic bacteria, and risk factors in patients with intracranial infections secondary to brain tumour surgery.

**Methods:**

A total of 208 patients admitted to our hospital for brain tumour surgery from January 2020 to May 2022 were selected. Fully automated biochemical analyzer was employed for CSF routine and for measuring biochemical parameters such as white blood cell (WBC), micrototal protein (M-TP), glucose (GLU), and chlorine (CI). Double antibody sandwich assay for CSF procalcitonin (PCT), heparin-binding protein (HBP), and matrix metalloproteinase-9 (MMP-9) was performed. Fully automated microbiological analyzer for pathogen identification was utilized. Based on the above results, we determined whether the patients had secondary intracranial infections after surgery and analyzed the risk factors for secondary intracranial infections after brain tumour surgery by univariate and multifactorial logistic regression.

**Results:**

Among 208 patients with brain tumour surgery, 65 cases (31.25%) had secondary intracranial infection and 143 cases (68.75%) had no secondary intracranial infection. The levels of WBC, M-TP, CI, PCT, HBP, and MMP-9 in the CSF of intracranially infected patients were significantly higher than those of uninfected patients (*P* < 0.05), and GLU was significantly lower than that of uninfected patients (*P* < 0.05), and the levels of PCT, HBP, and MMP-9 in infected patients were significantly lower than those before treatment after 3, 7, and 10 d and tended to decrease over time (*P* < 0.05). A total of 62 pathogenic strains were isolated from 65 intracranial infections, of which 41 (66.13%) were Gram-negative bacteria, mainly resistant to amikacin and ciprofloxacin and sensitive to meropenem and imipenem; 19 (30.65%) were Gram-positive bacteria, mainly highly resistant to penicillin and erythromycin and sensitive to vancomycin. Univariate analysis showed that age, gender, tumour type, history of glucocorticoid application, and prophylactic application of antibiotics were not associated with secondary intracranial infection after brain tumour surgery (*P* > 0.05); tumour site, operation time, postoperative indwelling drainage time, postoperative cerebrospinal fluid leakage, and history of diabetics were all associated with secondary intracranial infection after brain tumour surgery (*P* < 0.05). Multivariate logistic regression analysis showed that infratentorial tumour, operation time ≥4 h, postoperative indwelling drainage time ≥24 h, and postoperative cerebrospinal fluid leakage were independent risk factors for secondary intracranial infection after brain tumour surgery (*P* < 0.05).

**Conclusion:**

Patients with intracranial infections secondary to brain tumour surgery have abnormal levels of CSF routine and biochemical parameters, and the detection rate of Gram-negative bacteria is higher than that of Gram-positive bacteria in patients. Treatment should be based on the characteristics of pathogenic bacteria and risk factors with targeted interventions to reduce intracranial infections.

## 1. Introduction

Brain tumours are the most common intracranial tumours in neurosurgery, accounting for approximately 45% of all intracranial tumours, with slightly more men than women [1]. In the early stages of brain tumour development, patients may suffer from dizziness and headache, speech disturbances, visual disturbances, smell disturbances, hearing loss, unilateral limb numbness, unsteady walking, seizures, or pituitary problems, which are best treated by complete surgical resection [2–4]. However, in the postoperative period, the defensive effect of the blood-brain barrier system and the severe damage to the peripheral tissues of the patient's body make it easy for pathogenic bacteria to invade the cranial tissues and induce secondary intracranial infection, commonly occurring from 3 d to 7 d after surgery (10 d to 12 d after surgery in patients with drainage is the high incidence). It is one of the main causes of prolonged hospital stay, increased disability and mortality, and poor prognosis. Most patients with intracranial infection can be cured by anti-infection treatment, but the patient has an acute onset, accompanied by high fever and meningeal irritation. If the anti-infection treatment is not timely, the patient is still prone to brain injury sequelae and even death [5]. Cerebrospinal fluid (CSF) cultures are the gold standard for the diagnosis of intracranial infections. However, the low culture positivity rate of CSF pathogens, the relatively time-consuming drug sensitivity tests, and the waiting for the results before treatment will definitely delay the disease, coupled with the general increase in drug resistance of pathogenic bacteria, and the empirical use of antibacterial drugs cannot achieve satisfactory results, making the treatment of patients more difficult. CSF routine and biochemical tests are important for the diagnosis, differentiation, and assessment of intracranial diseases. Several studies [6, 7] have shown that white blood cell (WBC) counts in the CSF are elevated in patients with intracranial infections, but with lower sensitivity and specificity, and the measurement of micrototal protein (M-TP), glucose (GLU), and chloride (CI) can further strengthen the diagnosis and differentiation. In addition, in infectious diseases or sepsis, serum levels of infection indicators such as procalcitonin (PCT), heparin-binding protein (HBP), and matrix metalloproteinase-9 (MMP-9) are significantly increased [8,9], but the value of changes in PCT, HBP, and MMP-9 levels in CSF for the diagnosis of intracranial infections remains to be investigated. This study analyzed the CSF routine biochemical levels, pathogenic bacteria distribution, and risk factors in patients with secondary intracranial infections after brain tumour surgery, aiming to provide relevant reference for clinical diagnosis and prevention of secondary intracranial infections after brain tumour surgery.

## 2. Materials and Methods

### 2.1. Research Object

A total of 208 patients admitted to our hospital for brain tumour surgery from January 2020 to May 2022 were selected. Our goal is to determine whether the patient had a secondary intracranial infection after surgery by CSF routine and biochemical tests and identification of pathogenic bacteria. Diagnosis of intracranial infection was based on the following: (1) the patient's body temperature continued to rise, >38°C, and there were symptoms or signs such as fatigue, high fever, headache, vomiting, and meningeal irritation; (2) the bacterial culture of intracranial drainage fluid or CSF was positive; and (3) routine and biochemical tests of CSF samples suggested that WBC count in CSF increases; CSF turbidity and M-TP quantitatively increase; or GLU quantitatively decrease, etc., and intracranial infection could be determined if the above one was met [10]. Inclusion criteria were as follows: (1) if they met the diagnostic criteria of the National Institute for Health and Clinical Excellence (NICE) for brain tumours [11]; (2) age >18 years, complete case history, treated with brain tumour resection and meeting the appropriate indications [11], and expected survival time >7 d after surgery; and (3) those without consciousness impairment and voluntarily participated in the trial. Exclusion criteria were as follows: (1) death during or within 3 d after brain tumour surgery; (2) preoperative acute and chronic systemic or local infection; (3) those who had received preoperative medication such as glucocorticoids or antibiotics; (4) combined cranial hematoma, vascular disease, cranial abscess, cranial parasitic disease, soft tissue infection of the head, etc.; and (5) people with cardiac insufficiency and liver and kidney dysfunction.

### 2.2. Research Methods

#### 2.2.1. Cerebrospinal Fluid Detection

A lumbar puncture was performed to collect 10 ml of CSF from the patients 2 d after surgery (or at the onset of symptoms such as fever and meningeal irritation and so on) and on the 3, 7, and 10 days after drug treatment. AU5800 fully automatic biochemical analyzer (Beckman Coulter Inc) was employed for CSF routine and for measuring biochemical parameters such as WBC, M-TP, GLU, and CI levels. Double antibody sandwich assay was carried out for the determination of PCT, HBP, and MMP-9 levels in CSF, and the kits were purchased from Abcam. Vitek32 fully automated microbiological analyzer (BioMérieux, France) was utilized for pathogen identification. Based on these findings, we determined whether the patient had a secondary intracranial infection after surgery.

#### 2.2.2. Data Collection

The clinical data of patients were collected by querying electronic cases, including age, sex, tumour type, tumour site, operation time, postoperative indwelling drainage time, postoperative cerebrospinal fluid leakage, history of diabetics, history of glucocorticoid application, and prophylactic application of antibacterial drugs. The case data collected were entered and checked centrally using EpiData, which was required to ensure the accuracy of the data in the entry process.

#### 2.2.3. Drug Treatment

Treat with broad-spectrum antibacterial drugs or select sensitive antibacterial drugs based on the patient's drug sensitivity test results. Antibacterial drugs that could cross the blood-brain barrier such as amikacin, meropenem, and vancomycin should be chosen.

### 2.3. Statistical Analysis

SPSS 22.0 was employed for statistical analysis. The measurement data were expressed as $\bar{x\pm s}$, and independent samples *t*-test was performed for both groups. Statistical data were described in terms of cases and percentages (%), with the *χ*^2^ test. Univariate analyses that were statistically significant were included in multivariate analyses, and risk factors for secondary intracranial infection after brain tumour surgery were analyzed by multivariate logistic regression. *P* > 0.05 was considered statistically significant.

## 3. Result

### 3.1. Postoperative Infections in 208 Patients Operated for Brain Tumours

**Figure 1:**
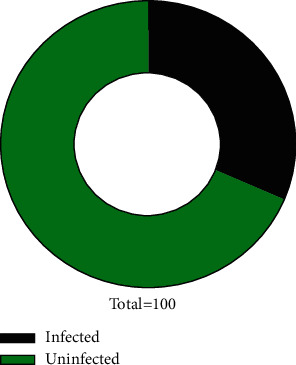
Pie chart of postoperative infections in 208 patients operated for brain tumours.

Among 208 patients with brain tumour surgery, 65 cases (31.25%) had secondary intracranial infection and 143 cases (68.75%) had no secondary intracranial infection (Table 1, Figure 1).

### 3.2. Analysis of CSF Routine and Biochemical Parameters in 208 Patients Operated for Brain Tumors

**Figure 2:**
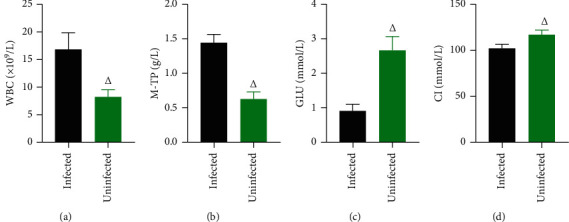
Analysis of CSF routine and biochemical parameters in 208 patients operated for brain tumours. *Note*. Comparison with the same indicator in infected persons, ^Δ^*P* < 0.05.

The levels of WBC, M-TP, and CI in the CSF of intracranially infected patients were significantly higher than those of uninfected patients (*P* < 0.05), and GLU was significantly lower than that of uninfected patients (*P* < 0.05). (Figure 2).

### 3.3. Analysis of PCT, HBP, and MMP-9 Levels in CSF of 208 Patients Operated for Brain Tumours

**Figure 3:**
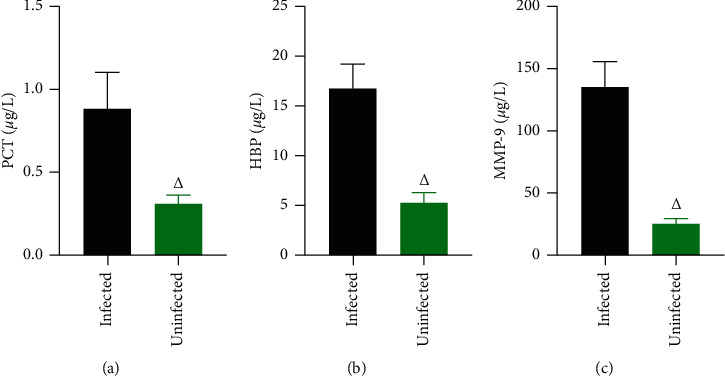
Analysis of PCT, HBP, and MMP-9 levels in CSF of infected and uninfected patients (*μ*g/L). *Note*. Comparison with the same indicator in infected persons, ^Δ^*P* < 0.05.

**Figure 4:**
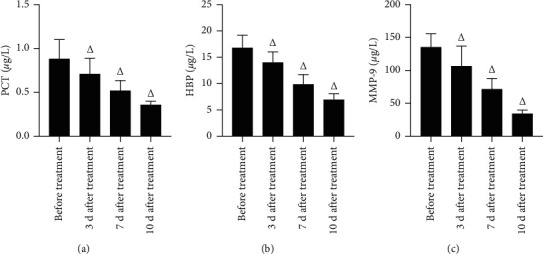
Analysis of PCT, HBP, and MMP-9 levels before and after treatment in 65 patients with intracranial infections (*μ*g/L). *Note*. Comparison with the same indicator before treatment, ^Δ^*P* < 0.05.

The levels of PCT, HBP, and MMP-9 in the CSF of intracranially infected patients were significantly higher than those of uninfected patients (*P* < 0.05), and the levels of PCT, HBP, and MMP-9 in infected patients were significantly lower than those before treatment after 3, 7, and 10 d and tended to decrease over time (*P* < 0.05). (Figures 3 and 4).

### 3.4. Analysis of the Pathogenic Bacteria Distribution and Drug Resistance Rate in 65 Cases of Intracranial Infections

A total of 62 pathogenic strains were isolated from 65 intracranial infections, of which 41 (66.13%) were Gram-negative bacteria, mainly resistant to amikacin and ciprofloxacin and sensitive to meropenem and imipenem; 19 (30.65%) were Gram-positive bacteria, mainly highly resistant to penicillin and erythromycin and sensitive to vancomycin (Tables 2–4).

### 3.5. Single Factor Analysis of Intracranial Infection Secondary to Brain Tumour Surgery

Univariate analysis showed that age, gender, tumour type, history of glucocorticoid application, and prophylactic application of antibiotics were not associated with secondary intracranial infection after brain tumour surgery (*P* > 0.05); tumour site, operation time, postoperative indwelling drainage time, postoperative cerebrospinal fluid leakage, and history of diabetics were all associated with secondary intracranial infection after brain tumour surgery (*P* < 0.05). (Table 5).

### 3.6. Multifactorial Logistic Regression Analysis of Intracranial Infections Secondary to Brain Tumour Surgery

Multivariate logistic regression analysis showed that infratentorial tumour, operation time ≥4 h, postoperative indwelling drainage time ≥24 h, and postoperative cerebrospinal fluid leakage were independent risk factors for secondary intracranial infection after brain tumour surgery (*P* < 0.05). (Table 6).

## 4. Conclusion

During craniotomy, patients may present with clinical symptoms similar to those of intracranial infections due to irritation by the implant or other factors, making the differential diagnosis of intracranial infections difficult. CSF's laboratory-related tests are of great interest because of their ease of operation and rapidity of detection. The results of CSF routine and biochemical parameters in this study showed that the levels of WBC, M-TP, and CI in the CSF of those with intracranial infection secondary to brain tumour surgery were significantly higher than those of without infection, and the GLU was significantly lower than those of without infection. This suggests that CSF routine and biochemical tests can go some way to diagnosing intracranial infections. Nevertheless, as intracranial infections have many causes, the main common ones being bacterial, viral, and tuberculosis infections [12, 13], this study further analyzed the markers of infection in the CSF of patients after brain tumour surgery. The results found that the levels of PCT, HBP, and MMP-9 in the CSF of those with intracranial infection were significantly higher than those of without infection. It is suggested that PCT, HBP, and MMP-9 tests are potentially valuable in the early diagnosis of intracranial infection after brain tumour surgery. PCT is a common biological indicator for the diagnosis of bacterial infections and sepsis and is now recognized to be significantly elevated in bacterial infections combined with a systemic inflammatory response, and its elevation correlates with the severity of the patient's infection, whereas in viral infections or aseptic inflammation, PCT is normal or mildly elevated [14]. Studies [15, 16] found that the PCT levels of serum and cerebrospinal fluid in patients with intracranial infection on the first day after craniotomy were significantly higher than those in noninfected patients, and their sensitivity and specificity in the diagnosis of intracranial infection were more than 80%. HBP is an acute reactive protein with the function of regulating macrophages and mediating inflammatory response. In the early stage of infection, endotoxin of pathogenic bacteria stimulates neutrophils to release a large amount of HBP, which leads to acute inflammatory response, resulting in damage to blood-brain barrier and increased permeability. It has been pointed out [17] that when the level of cerebrospinal fluid HBP is >11.84 *μ*g/L, the sensitivity of its differential diagnosis between acute bacterial meningitis and viral meningitis can reach more than 90%. MMP-9 is a novel marker of infection that relies on the assistance of metal ions such as Ca^2+^ and Zn^2+^ to exert extracellular matrix degradation [18]. Animal studies [19] have shown that when the content of MMP-9 in the blood increases, the vascular basement membrane and blood-brain barrier are damaged, and inflammatory cells enter the cerebral vessels, resulting in angiogenic brain edema, which is prone to intracranial infection. In this study, PCT, HBP, and MMP-9 levels were significantly lower than before treatment after 3, 7 and 10 d in those with intracranial infection secondary to brain tumour surgery and tended to decrease over time. It indicates that PCT, HBP, and MMP-9 can be used for early assessment of the extent of intracranial infections and to guide rational clinical management.

Brain tumours occur in a confined environment formed by the tissues of the meninges, skull, and scalp, and when patients need to undergo craniotomy, the disruption of the anatomical structure caused by the surgery and the placement of postoperative drains can open up the brain tissue to the outside world; thus, causing germs to invade the skull and increasing the probability of infection. CSF pathogenic cultures are the gold standard for the diagnosis of intracranial infections, and their epidemiological surveillance is important in guiding the empirical clinical use of drugs. In this study, 62 strains of pathogenic bacteria were isolated from 65 cases of intracranial infections, of which Gram-negative bacteria accounted for 66.13%, with Acinetobacter baumannii and *Klebsiella pneumoniae* being the most distributed, and these two bacteria were mainly highly resistant to amikacin and ciprofloxacin and sensitive to meropenem and imipenem, so we presume that intracranial infections caused by Gram-negative bacteria should be treated with beta-lactam antibiotics; Gram-positive bacteria accounted for 30.65%, with coagulase-negative staphylococci and *Staphylococcus aureus* being the most distributed, and these two bacteria were mainly highly resistant to penicillin and erythromycin and sensitive to vancomycin, suggesting that vancomycin could be the antibacterial drug of choice for intracranial infection caused by Gram-positive bacteria.

Intracranial infection after craniocerebral surgery often coexists with encephalocele, intracerebral hypertension, brain edema, and hydrocephalus, and they interact with each other to aggravate the condition; thus, directly affecting the prognosis. At present, it is believed that there are some differences in the related factors of secondary intracranial infection after brain tumour surgery at home and abroad. In this study, multivariate logistic regression was used to adjust for confounding factors. It was found that the independent risk factors of intracranial infection after brain tumour surgery include the following: first, infratentorial tumour: according to the report [20], the incidence of intracranial infection in infratentorial craniotomy is 6 times higher than that in supratentorial craniotomy, which may be due to the complex anatomical structure of the infratentorial posterior cranial fossa, difficult exposure of the surgical site, and long operation time of microscope and other equipment, which can increase the risk of intracranial infection; also, the surgical approach of the posterior cranial fossa is adjacent to the mastoid air chamber, and the sudden opening of the mastoid air chamber during craniotomy will also increase the risk factor of intracranial infection. Secondly, operation time ≥4 h: craniocerebral surgery requires strict asepsis, and indoor air is an important source of pollution in the occurrence of intracranial infection; the more 10000 to 20000 bacteria fall into the surgical field per hour during surgery, the longer the operation time, the higher the probability of pollution exposure, and the higher the probability of postoperative infection [21]. Thirdly, postoperative indwelling drainage time ≥24 h: brain tumour surgery often requires the placement of intracranial or subcutaneous drains to facilitate the smooth drainage of cerebrospinal fluid or blood, but improper management of the drainage device or overpositioning of the drainage tube can result in poor drainage or even reflux of drainage fluid into the skull.

Brain tumour surgery often requires intracranial or subcutaneous indwelling drainage tubes in order to successfully drain cerebrospinal fluid or hematocele, but if the drainage device is not managed properly and the position of the drainage tube is too high, it can lead to poor drainage and even the drainage fluid flowing back to the brain; in addition, the risk of pollution exposure is correspondingly increased due to the long time of tube placement. Fourthly, postoperative cerebrospinal fluid leakage: cerebrospinal fluid leakage can be divided into rhinorrhea and otorrhea, and pathogenic bacteria can enter the brain along the channel of cerebrospinal fluid leakage, resulting in intracranial infection. It can be seen from the above that for patients with brain tumour surgery, adequate preparation and detailed operation plan should be made before operation, the operation time should be shortened as far as possible, aseptic operation should be strictly implemented, the wound should be closely sutured after operation, cerebrospinal fluid leakage should be prevented, drainage tubes should be placed to avoid reflux and blockage caused by improper placement, and drainage time should also be controlled. For patients with brain tumour surgery with high-risk factors, prophylactic drugs should be used and monitoring should be strengthened during the perioperative period. Once there are suspected symptoms of intracranial infection such as high fever and meningeal irritation, diagnosis and treatment should be carried out as soon as possible without any delay.

In summary, patients with intracranial infections secondary to brain tumour surgery have abnormal levels of CSF routine and biochemical parameters, and the detection rate of Gram-negative bacteria is higher than that of Gram-positive bacteria in patients. Treatment should be based on the characteristics of pathogenic bacteria and risk factors with targeted interventions to reduce intracranial infections. This study also has shortcomings. There are many factors that contribute to cerebrospinal fluid infection after craniotomy, and the clinical symptoms of each type of infection lack specificity. Studying the routine and biochemistry of the cerebrospinal fluid in different types of infections may further strengthen the diagnosis and differentiation. However, given the inadequate sample size and technical support in this study, further classification analysis is pending for future studies with larger samples.

## Figures and Tables

**Table 1:** Postoperative infections in 208 patients operated on for brain tumours.

Infection status	*n*	Percentage
Infected	65	31.25
Uninfected	143	68.75

**Table 2:** Analysis of the pathogenic bacteria distribution in 65 cases of intracranial infections.

Pathogenic bacteria	Number	Percentage (%)
Gram-negative bacteria	41	66.13
*Acinetobacter baumannii*	13	20.97
*Klebsiella pneumoniae*	13	20.97
Pseudomonas aeruginosa	9	14.51
Escherichia coli	6	9.68
Gram-positive bacteria	19	30.65
Coagulase-negative staphylococci	7	11.29
*Staphylococcus aureus*	6	9.68
*Staphylococcus epidermidis*	3	4.84
*Enterococcu*s	3	4.84
Fungus	2	3.22
Candida albicans	2	3.22
Total	62	100.00

**Table 3:** Resistance rates of major Gram-negative bacteria to antibacterial drugs.

Antibacterial drug	Acinetobacter baumannii (*n* = 13)		*Klebsiella pneumoniae* (*n* = 13)	
	Number	Resistance rate (%)	Number	Resistance rate (%)
Amikacin	11	84.62	11	84.62
Ciprofloxacin	13	100.00	8	61.54
Gentamicin	10	76.92	7	53.85
Ceftazidime	10	76.92	8	61.54
Cefuroxime	9	69.23	8	61.54
Piperacillin	6	46.15	6	46.15
Meropenem	0	0.00	0	0.00
Imipenem	0	0.00	0	0.00

**Table 4:** Resistance rates of major Gram-positive bacteria to antibacterial drugs.

Antibacterial drug	Coagulase-negative staphylococci (*n* = 7)		*Staphylococcus aureus* (*n* = 6)	
	Number	Resistance rate (%)	Number	Resistance rate (%)
Penicillin	7	100.00	6	100.00
Erythromycin	7	100.00	6	100.00
Oxacillin	5	71.43	5	83.33
Levofloxacin	5	71.43	3	50.00
Vancomycin	0	0.00	0	0.00
Rifampicin	1	14.29	1	16.67

**Table 5:** Univariate analysis of intracranial infection secondary to brain tumour surgery.

Risk factor	Infected (*n* = 65)		Uninfected (*n* = 143)		*χ* ^2^	*P*
	*n*	Percentage	*n*	Percentage		
Age (years)					2.239	0.327
<35	10	15.38	35	24.47		
3535∼<60	24	36.92	45	31.47		
≥60	31	47.70	63	44.06		
Gender					0.009	0.925
Male	35	53.85	78	54.55		
Female	30	46.15	65	45.45		
Tumour type					6.182	0.103
Meningioma	25	38.46	40	27.97		
Glioma	23	35.39	66	46.15		
Pituitary adenoma	6	9.23	23	16.09		
Acoustic nerve tumour	11	16.92	14	9.79		
Tumour site					15.820	<0.001
Supratentorial	32	49.23	110	76.92		
Infratentorial	33	50.77	33	23.08		
Operation time (h)					21.083	<0.001
<4	15	23.08	82	57.34		
≥4	50	76.92	61	42.66		
Postoperative indwelling drainage time					20.302	<0.001
Not put or <24 (h)	25	38.46	102	71.33		
≥24	40	61.54	41	28.67		
Postoperative cerebrospinal fluid leakage					17.033	<0.001
Yes	52	80.00	71	49.65		
No	13	20.00	72	50.35		
History of diabetes					7.608	0.006
Yes	19	29.23	19	13.29		
No	46	70.77	124	86.71		
History of glucocorticoid application					0.002	0.966
Yes	17	26.15	37	25.87		
No	48	73.85	106	74.13		
Prophylactic application of antibiotics					0.573	0.449
Yes	25	38.46	63	44.06		
No	40	61.54	80	55.94		

**Table 6:** Multifactorial logistic regression analysis of intracranial infections secondary to brain tumour surgery.

Risk factor	*β*	SE	Wald	OR	95% CI	*P*
Infratentorial tumour	1.946	0.665	8.514	7.001	1.901∼25.775	0.004
Operation time ≥4 h	1.015	0.328	9.790	2.759	1.451∼5.248	0.002
Postoperative indwelling drainage time ≥24 h	0.725	0.303	5.768	2.065	1.140∼3.739	0.018
Postoperative cerebrospinal fluid leakage	0.844	0.248	11.420	2.306	1.430∼3.781	0.001

## Data Availability

The data used in the current study are available from the corresponding author.

## References

[1] Higashiyama A., Matsuki M. (2021). Brain tumor. *No Shinkei Geka*.

[2] Shooli H., Nemati R., Ahmadzadehfar H. (2021). Theranostics in brain tumors. *PET Clinics*.

[3] Zhao R., Xiang J., Wang B., Chen L., Tan S. (2022). Recent advances in the development of noble metal NPs for cancer therapy. *Bioinorganic Chemistry and Applications*.

[4] Chen D. Y., Chen C. C., Crawford J. R., Wang S. G. (2018). Tumor-related epilepsy: epidemiology, pathogenesis and management. *Journal of Neuro-Oncology*.

[5] Ganjeifar B., Morshed S. F. (2021). Targeted drug delivery in brain tumors-nanochemistry applications and advances. *Current Topics in Medicinal Chemistry*.

[6] Yu M., Miao J., Lv Y. (2020). A challenging diagnosis of atypical glut1-DS: a case report and literature review. *Frontiers in Neurology*.

[7] Zhai T., Fu Z. L., Qiu Y. B., Chen Q., Luo D., Chen K. (2020). Application of combined cerebrospinal fluid physicochemical parameters to detect intracranial infection in neurosurgery patients. *BMC Neurology*.

[8] Venge P., Eriksson S., Pauksen K. (2021). Blood biomarker algorithms for the diagnosis of mycoplasma pneumoniae respiratory infections. *Journal of Immunological Methods*.

[9] Ren D., Wu D., Liu F., Jiao S., Wu Y. (2021). Diagnostic value of heparin-binding protein in the cerebrospinal fluid for purulent meningitis in children. *Brazilian Journal of Medical and Biological Research*.

[10] Ministry of Health of the People’s Republic of China (2001). Diagnostic criteria for hospital infections (trial). *Chinese Medical Journal*.

[11] Bates A., Gonzalez-Viana E., Cruickshank G., Roques T., Guideline Committee (2018). Primary and metastatic brain tumours in adults: summary of NICE guidance. *BMJ*.

[12] Martin A., Winn A., Sanchez A., Castellon I., Munera F., Nunez D. (2020). MRI of emergent intracranial infections and their complications. *Topics in Magnetic Resonance Imaging*.

[13] Wang Y., Liu Y., Chen R., Qiao L. (2021). Metabolomic characterization of cerebrospinal fluid from intracranial bacterial infection pediatric patients: a pilot study. *Molecules*.

[14] Han J., Gatheral T., Williams C. (2020). Procalcitonin for patient stratification and identification of bacterial co-infection in COVID-19. *Clinical Medicine*.

[15] Yu Y., Li H. J. (2017). Diagnostic and prognostic value of procalcitonin for early intracranial infection after craniotomy. *Brazilian Journal of Medical and Biological Research*.

[16] Zhu L., Dong L., Li Y. (2019). The diagnostic and antibiotic reference values of procalcitonin for intracranial infection after craniotomy. *World Neurosurgery*.

[17] Huang C. Z., Zhang J., Mo L. Y. (2019). Value of cerebrospinal fluid heparin-binding protein assay in pediatric septic meningitis. *Chinese Journal of Laboratory Medicine*.

[18] Chang M., Nguyen T. T. (2021). Strategy for treatment of infected diabetic foot ulcers. *Accounts of Chemical Research*.

[19] Cao S., Zhu P., Yu X. (2016). Hydrogen sulfide attenuates brain edema in early brain injury after subarachnoid hemorrhage in rats: possible involvement of MMP-9 induced blood-brain barrier disruption and AQP4 expression. *Neuroscience Letters*.

[20] Jeong T. S., Yee G. T. (2018). Prospective multicenter surveillance study of surgical site infection after intracranial procedures in korea: a preliminary study. *Journal of Korean Neurosurgical Society*.

[21] Dai B., Hu Z. Q., Zhu G. T. (2019). Influencing factors for intracranial infection in patients with craniocerebral tumours and etiological characteristics of cerebrospinal fluid. *Chinese Journal of Hospital Infection*.

